# Using Consistently Low Performance to Identify Low-Quality Physician Groups

**DOI:** 10.1001/jamanetworkopen.2021.17954

**Published:** 2021-07-28

**Authors:** Christina A. Nguyen, Lauren G. Gilstrap, Michael E. Chernew, J. Michael McWilliams, Bruce E. Landon, Mary Beth Landrum

**Affiliations:** 1 Massachusetts Institute of Technology, Cambridge; 2 Department of Health Care Policy, Harvard Medical School, Boston, Massachusetts; 3 Heart and Vascular Center, Dartmouth-Hitchcock Medical Center, Lebanon, New Hampshire; 4 Dartmouth Institute for Health Policy and Clinical Practice, Geisel School of Medicine at Dartmouth, Hanover, New Hampshire; 5 Division of General Internal Medicine and Primary Care, Department of Medicine, Brigham and Women’s Hospital, Boston, Massachusetts; 6 Division of General Internal Medicine, Beth Israel Deaconess Medical Center, Boston, Massachusetts

## Abstract

**Importance:**

There has been a growth in the use of performance-based payment models in the past decade, but inherently noisy and stochastic quality measures complicate the assessment of the quality of physician groups. Examining consistently low performance across multiple measures or multiple years could potentially identify a subset of low-quality physician groups.

**Objective:**

To identify low-performing physician groups based on consistently low performance after adjusting for patient characteristics across multiple measures or multiple years for 10 commonly used quality measures for diabetes and cardiovascular disease (CVD).

**Design, Setting, and Participants:**

This cross-sectional study used medical and pharmacy claims and laboratory data for enrollees ages 18 to 65 years with diabetes or CVD in an Aetna health insurance plan between 2016 and 2019. Each physician group’s risk-adjusted performance for a given year was estimated using mixed-effects linear probability regression models. Performance was correlated across measures and time, and the proportion of physician groups that performed in the bottom quartile was examined across multiple measures or multiple years. Data analysis was conducted between September 2020 and May 2021.

**Exposures:**

Primary care physician groups.

**Main Outcomes and Measures:**

Performance scores of 6 quality measures for diabetes and 4 for CVD, including hemoglobin A_1c_ (HbA_1c_) testing, low-density lipoprotein testing, statin use, HbA_1c_ control, low-density lipoprotein control, and hospital-based utilization.

**Results:**

A total of 786 641 unique enrollees treated by 890 physician groups were included; 414 655 (52.7%) of the enrollees were men and the mean (SD) age was 53 (9.5) years. After adjusting for age, sex, and clinical and social risk variables, correlations among individual measures were weak (eg, performance-adjusted correlation between any statin use and LDL testing for patients with diabetes, *r* = −0.10) to moderate (correlation between LDL testing for diabetes and LDL testing for CVD, *r* = .43), but year-to-year correlations for all measures were moderate to strong. One percent or fewer of physician groups performed in the bottom quartile for all 6 diabetes measures or all 4 cardiovascular disease measures in any given year, while 14 (4.0%) to 39 groups (11.1%) were in the bottom quartile in all 4 years for any given measure other than hospital-based utilization for CVD (1.1%).

**Conclusions and Relevance:**

A subset of physician groups that was consistently low performing could be identified by considering performance measures across multiple years. Considering the consistency of group performance could contribute a novel method to identify physician groups most likely to benefit from limited resources.

## Key Points

**Question:**

Can quality measures identify low-quality physician groups when performance is correlated across multiple measures or multiple years?

**Findings:**

In this cross-sectional study of a commercially insured population with diabetes or cardiovascular disease, we found weak consistency of low performance scores across multiple measures but moderate to strong consistency of scores over multiple years. One percent or fewer of physician groups performed in the bottom quartile for all diabetes measures or all cardiovascular disease measures in any given year, while 4% to 11% were in the bottom quartile in all 4 years for most measures.

**Meaning:**

These results suggest that consistency in poor performance depends on the statistical properties of the measures.

## Teaser

This cross-sectional study assesses the use of consistently low performance scores for physician groups across multiple performance measures and over multiple years to identify low-quality physician groups.

## Introduction

In the past decade, there has been a shift away from fee-for-service and toward population-based payment models that reward physician groups based on performance on quality measures. However, the multidimensionality and stochastic nature of quality measures may complicate assessment and, more specifically, the identification of inadequately performing physician groups. In particular, groups may be incorrectly identified as poor performing purely by chance in common forms of cross-sectional analyses.

In addition, the burden of quality measurement on physicians and the substantial investments made in measurement development have been a continuous concern.^1,2,3,4^ In the US, physician practices spend more than $15.4 billion annually on reporting quality measures alone.^4^ With health care expenditures rising, it is increasingly critical that resources for tracking quality are used effectively.

Previous work has assessed consistency in quality measurement in hospitals and health plans. Various national organizations and payers have examined performance across multiple measures to rate hospitals and also explored whether hospitals are ranked differently from 1 year to another.^5,6,7,8,9,10,11,12^ Other analyses have investigated the quality of health plans using the Healthcare Effectiveness Data and Information Set (HEDIS), finding variation and lack of correlation among performance measures.^13,14^

Previous research has also focused on hospital or health plan quality performance and has mostly studied variation and changes in ranking (eg, different ranking methods, ranking in different years), but there has been less work on identification of low performance. It is important to measure quality at the physician group level because, increasingly, groups are responsible for population-based outcomes. In addition, rankings are often not useful because of the inherent noisiness of measures.^15^ We propose the use of consistently low performance across multiple quality measures or multiple years as a method to identify low-performing physician groups.

In this study, we used medical and pharmacy claims and laboratory data from an Aetna health insurance plan to examine whether consistently low performance across multiple quality measures or multiple years can identify low-performing physician groups. We measured performance using commonly used measures for 2 common chronic conditions that have been the focus of many quality measurement and improvement efforts: diabetes and cardiovascular disease (CVD).

## Methods

### Study Data

We used medical claims, pharmacy claims, and laboratory results from an Aetna health insurance plan between 2016 and 2019. Our sample included adults between ages 18 and 65 years with either diabetes or CVD (based on 2013 HEDIS eligibility criteria: 1 or more inpatient visit or 2 or more outpatient visits with an *International Statistical Classification of Diseases and Related Health Problems, Tenth Revision *[*ICD-10*] code for diabetes or CVD) who were continuously enrolled for at least 1 calendar year between 2016 and 2019.

We attributed each enrollee to the physician group (defined by tax identification number [TIN]) accounting for the plurality of the enrollee’s office visits during a given year (see eAppendix 1 in the Supplement). We assigned enrollees with the same number of visits to multiple TINs to the one with the greatest total payments.^16^

To ensure sufficient precision in group-level estimates, we restricted our sample to groups with at least 40 attributed enrollees with diabetes and at least 40 with CVD, of which 20 enrollees had to have laboratory and pharmacy data for every relevant measure in a given year.

We obtained institutional review board approval from Harvard University’s Committee on the Use of Human Subjects. Informed consent was not required because data sets were deidentified. Analysis was conducted between September 2020 and May 2021. This article is compliant with the Strengthening the Reporting of Observational Studies in Epidemiology (STROBE) reporting guideline for cross-sectional studies.

### Study Variables

#### Quality Measures

We constructed 10 quality measures, including HEDIS-based process and disease control measures and outcome measures commonly used in the literature (see eAppendix 2 in the Supplement). For diabetes, we constructed 3 process measures (hemoglobin A_1c_ [HbA_1c_] testing, low-density lipoprotein [LDL] testing, and statin use [1 or more fill over the measurement year]), 2 disease control measures (HbA_1c_ control [less than 8%] and LDL control [below 100 mg/dL; to convert to millimoles per liter, multiply by 0.0259]), and 1 utilization-based outcome measure (no emergency department [ED] visit, observation stay, or hospital admissions for diabetes or major adverse cardiovascular events [MACE]^17^). For CVD, we included measures for LDL testing, statin use, LDL control, and no ED, observation, or hospital admissions for MACE. Some measures were limited to subsets of enrollees with relevant pharmacy benefits with the same insurer (42.7% for diabetes cohort [235 546 of 551 415 enrollees] and 41.2% for CVD cohort [291 597 of 708 171 enrollees]) and available laboratory results (54.3% for diabetes cohort [299 215 of 551 415] and 42.6% for CVD cohort [301 671 of 708 171]). The clinical and area-level characteristics of enrollees with and without laboratory or pharmacy data were similar (eTable 1 in the Supplement).

#### Covariates

Because patient characteristics are often associated with performance,^18,19,20,21,22,23,24,25^ we adjusted for both patient-level clinical and area-level social risk variables. Clinical covariates included a set of comorbidities (atrial fibrillation, hypertension, chronic obstructive pulmonary disease, heart failure, and chronic kidney disease) that were coded using the CMS Chronic Condition Warehouse definitions,^26^ and a comorbidity score based on a DxCG Intelligence version 5.0.0 (Cotiviti) prediction model. We used zip code–level sociodemographic characteristics from the 2010 US Census (percentage of population who were Black, Hispanic, and college educated) and 5-year estimates from the 2010-2014 American Community Survey (percentage of population below poverty). Using methods from a previous study, we also assigned enrollees to urban, suburban, and rural classifications based on zip code.^27^

### Statistical Analysis

We used inverse probability weighting to account for observed differences between enrollees with and without pharmacy or laboratory data. Specifically, to standardize the samples across measures that required laboratory or pharmacy data, we weighted each patient as the inverse of the probability that they had laboratory (for disease control) or pharmacy (for statin use) measures, respectively. These weights were estimated using a logit model that included all of the covariates described above and used the availability of data as the dependent variable for the relevant measures.

Because low-performing groups may be more likely to treat high-risk patients, we followed a 2-step social risk adjustment methodology from earlier studies^22,28,29^ for computing adjusted quality performance. Prior work has shown that social risk adjustment can affect the variance and rankings of physician group performance on disease control and outcome measures, but is less of a factor for process measures.^22^ For consistency, we adjusted all quality measures, including process measures. In this approach, we removed the association of high-risk patients sorting to low-performing physician groups by basing our adjustment on within-group associations. In the first step, we fit inverse probability weighted linear regression models estimating the adherence of an enrollee attributed to a physician group in a given year as a function of patient-level age and gender, patient-level comorbidities and DxCG composite, zip code–level sociodemographic characteristics, and physician group fixed effects. We then computed an enrollee-year–level risk score as the projected performance for each measure estimated from only the coefficients on the enrollee characteristics (ie, not including the coefficients on the group fixed effects in the estimate).

In the second step, we computed group-level performance scores. We estimated patient-level mixed-effects linear probability regression models that related the performance on a measure to the risk-score computed in step 1 and to physician group random effects. We then computed an adjusted score that represented a group’s estimated performance in a given year for an enrollee with average clinical and social risk. A more detailed explanation of this 2-step adjustment approach can be found in eAppendix 3 in the Supplement.

To estimate the degree of variation in performance at the physician group level, we computed intraclass correlation coefficients (ICCs). The ICC represents the fraction of total variation in performance that is explained by differences between physician groups. The ICC can be low if there is little variation between groups or if the within-group variance is high. Measures with low ICCs generally have less ability to distinguish performance at the group level and are thus less useful for identifying low-performing clinicians. We also computed reliability for each measure. Reliability represents a measure’s reproducibility and is a function of the measure of variation *within* and *between* physician groups, as well as the sample size. We report reliability for practices based on the median number of enrollees.

To examine consistency across measures and years, we computed pairwise and year-to-year (eg, 2016 vs 2017, 2017 vs 2018) correlations among physician groups’ risk-adjusted performance on measures. We tabulated the proportion of physician groups that performed in the bottom quartile of scores across multiple measures or multiple years. We defined low-performing physician groups as falling into the bottom quartile of quality performance. After excluding those that did not have complete data for all 4 years, 353 physician groups were included in the analysis of consistency in performance across years. All analyses were performed using Stata statistical software version 15.1 (StataCorp). *P* < .05 was treated as statistically significant, and all statistical tests were 2-sided.

## Results

Our final sample included 786 641 unique enrollees (1 189 367 enrollee-years, of which 481 196 were patients with diabetes only, 637 952 patients with CVD only, and 70 219 patients with both) treated by 890 physician groups (634 in 2016, 564 in 2017, 593 in 2018, and 558 in 2019). A total of 414 655 (52.7%) of enrollees were men and the mean (SD) age was 53 (9.5) years.

### Performance on Individual Measures

For diabetes, median (interquartile range [IQR]) rates of adjusted performance were high on HbA_1c_ testing (90.9% [89.2%-94.5%]), LDL testing (89.4% [86.9%-92.5%]), and avoidance of hospital-based utilization (78.9% [77.6%-80.3%]) (Table 1). Performance was lower on HbA_1c_ control (65.2% [62.9%-67.5%]), LDL control (66.4% [64.5%-68.3%]), and statin use (57.8% [56.5%-62.0%]). The IQRs, which illustrate the variability of adjusted performance across measures, were narrow: the difference between the upper and lower quartiles ranged from 2.7% for hospital-based utilization to 5.6% for LDL testing. Group-level variation explained a small proportion of the total variation in performance on the measures for diabetes: the ICCs, which indicate ability to distinguish performance, were lowest for hospital-based utilization (ranging from 0.6%-0.7% between 2016 and 2019) and highest for HbA_1c_ testing (4.7%-5.8%). Measure reliability was high for testing measures but moderate for hospital-based utilization for a group with the median number of attributed enrollees.

**Table 1.** Distribution of Adjusted Physician Group-Year Performance on Diabetes and Cardiovascular Disease Measures (N = 2349)

Measure	Median (IQR), %	Intraclass correlation coefficients, %^a^	Reliability, %
Diabetes			
HbA_1c_ testing	90.9 (89.2-94.5)	4.7-5.8	86.8-89.1
LDL testing	89.4 (86.9-92.5)	3.0-3.7	80.3-83.5
HbA_1c_ control^b^	65.2 (62.9-67.5)	1.0-1.5	56.1-66.3
LDL control^c^	66.4 (64.5-68.3)	0.6-1.0	45.4-58.1
Statin use	57.8 (56.5-62.0)	0.7-3.8	50.0-83.9
Hospital-based utilization	78.9 (77.6-80.3)	0.6-0.7	43.7-49.4
Cardiovascular disease			
LDL testing	79.3 (74.3-84.9)	3.1-4.3	84.9-88.6
LDL control^c^	40.3 (35.5-44.7)	0.5-1.4	47.8-71.1
Statin use	48.8 (41.1-59.6)	1.0-1.7	63.1-74.8
Hospital-based utilization	94.6 (92.2-96.4)	0.3-0.6	30.4-52.4

Abbreviations: HbA_1c_, hemoglobin A_1c_; IQR, interquartile range; LDL, low-density lipoprotein.

SI conversion: To convert LDL to millimoles per liter, multiply by 0.0259.

^a^ Intraclass correlation coefficients were computed from step 2 in the 2-step social risk adjustment approach, in which we estimated patient-level mixed-effects linear probability regression models that related performance in a given year on a measure to the risk-score computed in step 1 and physician group random effects.

^b^ HbA_1c_ control measures were less than 8%.

^c^ LDL control measures were below 100 mg/dL.

Similar to diabetes, median (IQR) rates of performance were high on LDL testing (79.3% [74.3%-84.9%]) and utilization-based outcomes (94.6% [92.2%-96.4%]) for CVD. Physician groups had lower performance on LDL control (40.3% [35.5%-44.7%]) and statin use (48.8% [41.1%-59.6%]). The IQR was lowest for hospital-based utilization (a range of 4.2%) and highest for statin use (18.5%). Again, ICCs were low; they were lowest for hospital-based utilization (0.3%-0.6%) and highest for LDL testing (3.1%-4.3%). Measure reliability was highest for testing and lowest for hospital-based utilization.

### Correlations Between Performance on Individual Measures

There were weak to moderate correlations between most of the individual quality measures, and only 4 of 45 pairwise correlations were greater than 0.5 (eTable 2 in the Supplement). Correlations were weak between different types of individual measures (eg, 0.01 between statin use and LDL testing for diabetes) and moderate between the same type of measures across the 2 disease cohorts (eg, *r* = 0.43 between LDL testing for diabetes and LDL testing for CVD). Testing measures had low correlations with their corresponding control measures. The highest positive correlation was in the CVD cohort between LDL control and statin use. Avoidance of hospital-based utilization was negatively correlated with LDL control and statin use for CVD.

We observed stronger consistency in performance on individual measures across years. Year-to-year correlations were highest for testing measures for both diabetes (mean *r* = 0.81 for HbA_1c_ testing and 0.68 for LDL testing) and CVD (mean *r* = 0.82 for LDL testing) (Table 2). They were lowest for LDL control for both diabetes (mean *r* = 0.51) and CVD (mean *r* = 0.46).

**Table 2.** Year-to-Year Correlations Between Adjusted Quality Performance on Diabetes and Cardiovascular Disease Measures Across Physician Groups

Measure	Correlation, *r*			
	2016 vs 2017	2017 vs 2018	2018 vs 2019	Mean
Diabetes				
HbA_1c_ testing	0.80	0.77	0.85	0.81
LDL testing	0.67	0.69	0.68	0.68
HbA_1c_ control^a^	0.53	0.52	0.60	0.55
LDL control^b^	0.51	0.55	0.48	0.51
Statin use	0.61	0.65	0.47	0.58
Hospital-based utilization	0.59	0.62	0.59	0.60
Cardiovascular disease				
LDL testing	0.79	0.86	0.82	0.82
LDL control^b^	0.44	0.45	0.48	0.46
Statin use	0.51	0.63	0.40	0.51
Hospital-based utilization	0.53	0.56	0.46	0.52

Abbreviations: HbA_1c_, hemoglobin A_1c_; LDL, low-density lipoprotein.

^a^ HbA_1c_ control measures were less than 8%.

^b^ LDL control measures were below 100 mg/dL.

### Consistency of Low Performance Across Multiple Measures or Multiple Years

**Figure 1.**
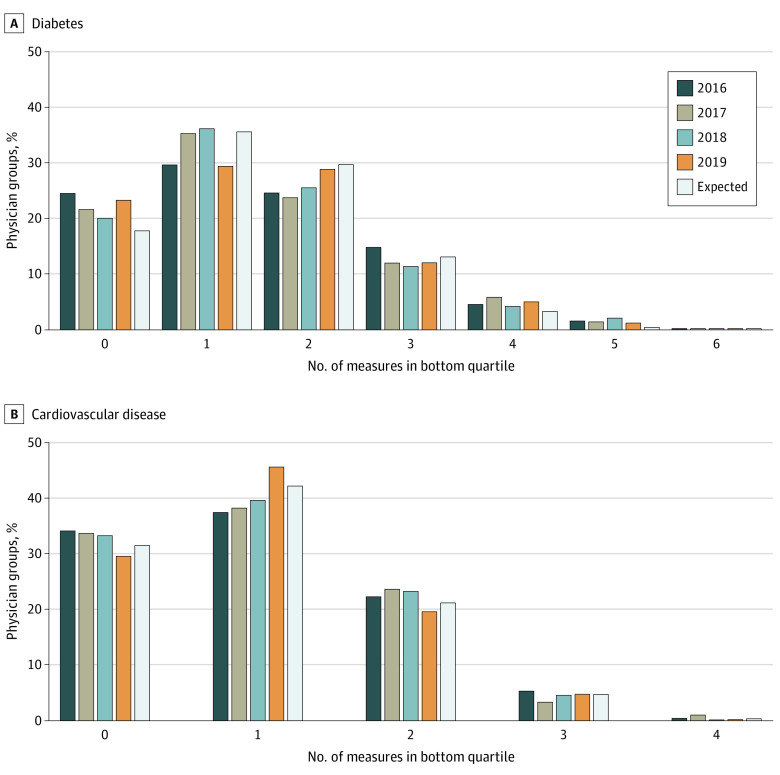
Consistency of Low Adjusted Performance Across Multiple Measures The expected bar is the proportion of physician groups expected to fall into the bottom quartile if performance on each measure in a given year was independent. For example, falling into the bottom quartile for 3 measures was computed as the probability of 3 success outcomes in 6 Bernoulli trials with a success probability of 0.25.

We observed minimal consistency in poor adjusted performance across multiple measures. Fewer than 0.2% of groups performed in the bottom quartile for all 6 diabetes measures in any given year (Figure 1). Similarly, 1% or fewer of groups performed in the bottom quartile for all 4 CVD measures in any given year.

Considering all 10 diabetes and CVD measures together, consistency in low performance remained weak (eFigure 1 in the Supplement). Fewer than 2% of groups were low performing on 7 or more measures. However, some groups could be flagged as consistently low quality based on testing and hospital-based utilization measures that had overall high performance and low variation across groups. The distinction of being in the bottom quartile for those measures was less meaningful. Although the variances for some of the control measures were low, we included them in the analysis because their mean performance was low to moderate and would thus be important to consider in quality assessment and improvement efforts. To examine consistency of low performance on potentially more meaningful measures, we removed HbA_1c_ testing for diabetes and LDL testing and hospital-based utilization for both diabetes and CVD. Including only disease control and statin use measures, about 1% or fewer were consistently low performing on all 5 (eFigure 2 in the Supplement).

**Figure 2.**
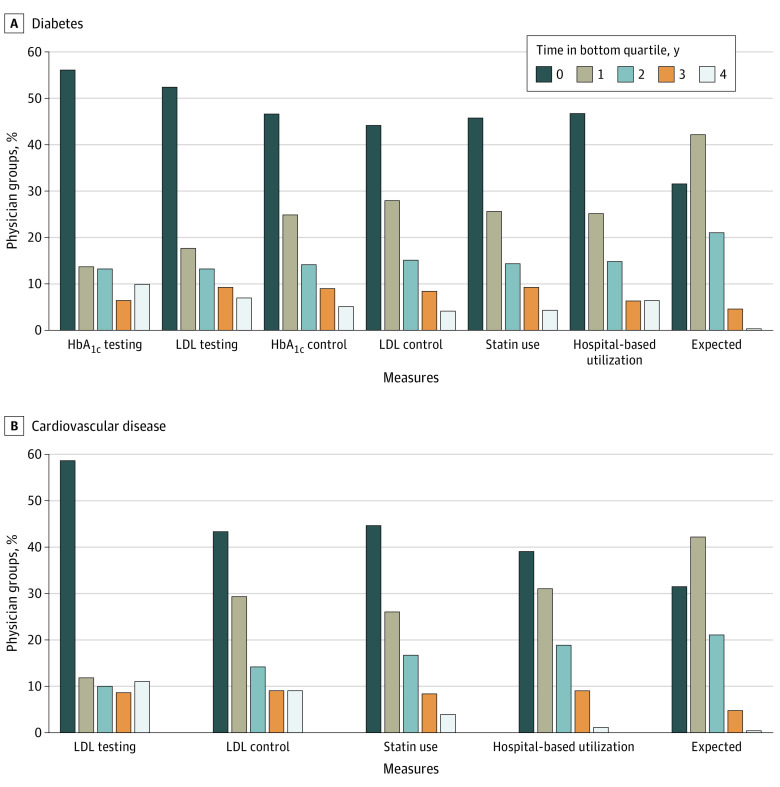
Consistency of Low Adjusted Performance Across Multiple Years The expected bar is the proportion of physician groups expected to fall into the bottom quartile if performance in each year for a given measure was independent. For example, falling into the bottom quartile for 3 years was computed as the probability of 3 success outcomes in 4 Bernoulli trials with a success probability of 0.25. HbA_1c_ indicates hemoglobin A1c; LDL, low-density lipoprotein.

We found higher consistency of low performance across multiple years (Figure 2). The percentage of groups that performed in the bottom quartile in all 4 years ranged from 25 (7.1%) to 39 groups (11.1%) for testing measures, 14 (4.0%) to 18 groups (5.1%) for disease control measures, 14 (4.0%) to 16 groups (4.5%) for statin use measures, and 4 (1.1%) to 23 groups (6.5%) for avoidance of hospital-based utilization measures. These rates were higher than the expected 0.4% if performance in each year was independent.

As expected, consistency in bottom quartile performance mirrored measure reliability. The percentage of groups that were consistently low performing (ie, falling into the bottom quartile in all 4 years) was highest for testing measures and lowest for hospital-based utilization, particularly in the CVD cohort. ICCs and reliability were lowest for hospital-based utilization measures (most out of physicians’ control).

In sensitivity analyses, we found that consistency of low performance across multiple measures or multiple years was similar if performance was unadjusted (ie, without controls for age, sex, and clinical and social risk factors) (eFigure 3 in the Supplement). In addition, a similar approach could be used to identify high-quality groups that focuses on consistency of high performance across multiple years (eFigure 4 in the Supplement).

## Discussion

In this study of physician groups caring for a commercially insured population with diabetes or CVD, we found that consistent low performance across multiple years could identify a subset of low-quality physician groups. Consistency in low performance across multiple measures was less useful in this setting. Our study expands previous research focused on hospital or health plan quality performance by examining performance at the physician group level and by introducing this novel, targeted approach to identify low-performing practices.

Variation across groups and measure reliability was highest among testing measures. Testing rates were generally high, particularly in the diabetes cohort. Measure reliability was low to moderate among potentially more clinically relevant measures of disease control, statin use, and avoidance of hospital-based utilization. Classifying a group as low performing using a single year of performance for these measures would thus identify groups as poor performing purely by chance. Examining performance across multiple years provides a method to identify poor performance on clinically relevant measures that cannot be reliably assessed in a single cross-section. Consistency across multiple measures could be useful in settings where individual measures are more correlated or to target groups with consistently poor performance in subsets of measures (eg, low rates of LDL testing for both diabetes and CVD). Pooling data across multiple years and/or creating composite measures are alternative approaches to improve precision in inherently noisy measures.^30,31,32,33,34,35,36^

As in previous studies,^13,37,38,39^ we observed low to moderate correlations between most individual measures. Although we did not find many groups that were consistently low performing across multiple measures, we did find a subset that were consistently low performing across multiple years. Rather than spreading efforts to track performance over a large number of practices, using this methodology to target a condensed number of underperforming groups could be the first line of defense against further declining or sustained low quality of care.^40,41^ Although all of the groups in this subset may not necessarily be low quality (because of statistical noise or unmeasured confounders), those that are truly low quality are most likely to be in this subset. Further scrutiny of these groups, perhaps through medical record reviews or site visits, could inform actionable initiatives that include financial incentives (or additional resources) or nonfinancial approaches to improve care. Similarly, high performers could be examined for best practices and possibly receive leniency on some data gathering requirements, allowing them to redirect some of their funds for data collection toward other areas of care.

### Limitations

This study has several limitations. First, the data came from 1 commercial health insurance plan that may have higher proportions of enrollees in certain regions of the US. This health plan may not cover a large portion of physician groups, and our quality metrics were computed based on a subset of each group’s patient panel. However, commercial data made it possible to examine quality in nonelderly populations and to construct disease control measures, which are typically uncommon in administrative data. Second, because we focused on quality of care for 2 chronic diseases, our analysis only included 10 measures. Third, following common practice, we used billing arrangements to identify physician groups. Fourth, because we did not have access to enrollee sociodemographic characteristics, we used zip code–level characteristics to adjust for social risk. Although we adjusted for both clinical (enrollee-level) and sociodemographic (area-level) characteristics, there may still be residual effects that we were unable to capture. Fifth, our results cannot be generalized to small practices, and to evaluate consistency of performance across multiple years we could only include physician groups with complete data across all 4 years. Sixth, we used relative thresholds to define poor performance. This approach is commonly used in value-based payments. However, performance classification can be sensitive to approach,^30,42^ and absolute thresholds may be more appropriate in some settings.^43^

## Conclusions

Moving forward with the use of quality performance to assess and reward health care professionals, it is important to consider the noisy nature of measures. In this article, we were able to identify a subset of physician groups based on their consistently low performance across multiple years. Consistency in performance could be applied to many other settings and could also be used to identify high-quality physicians. As quality measurement and incentives continue to be developed and are often directed at the organizational level, considering the consistency of group performance could afford a novel method to identify groups most likely to benefit from limited resources.

## 

## Notes

Supplement. **eAppendix 1.** Enrollee Attribution to Tax Identification Number (TIN) **eAppendix 2.** Quality Metric Coding **eAppendix 3.** Two-Step Social Risk Adjustment Methodology **eTable 1.** Characteristics of Enrollee-Years with Diabetes or Cardiovascular Disease by Availability of Laboratory and Pharmacy Data (%) **eTable 2.** Correlations Between Adjusted Performance on Quality Measures Across Physician Group-Years (N = 2,349) **eFigure 1.** Consistency of Low Adjusted Performance Across Multiple Diabetes and Cardiovascular Disease Measures **eFigure 2.** Consistency of Low Adjusted Performance Across Multiple Disease Control and Statin Use Measures for Diabetes and Cardiovascular Disease **eFigure 3.** Consistency of Low Unadjusted Performance Across Multiple Measures or Multiple Years for Diabetes and Cardiovascular Disease **eFigure 4.** Consistency of High Adjusted Performance Across Multiple Measures or Multiple Years for Diabetes and Cardiovascular Disease eReferences.
