# Supplementary material for: Using Consistently Low Performance to Identify Low-Quality Physician Groups
Source: JAMA Netw Open. 2021 Jul 28;4(7):e2117954. doi: 10.1001/jamanetworkopen.2021.17954 (PMC8319756; doi:10.1001/jamanetworkopen.2021.17954)
Supplement: Supplement. — eAppendix 1. Enrollee Attribution to Tax Identification Number (TIN) eAppendix 2. Quality Metric Coding eAppendix 3. Two-Step Social Risk Adjustment Methodology eTable 1. Characteristics of Enrollee-Years with Diabetes or Cardiovascular Disease by Availability of Laboratory and Pharmacy Data (%) eTable 2. Correlations Between Adjusted Performance on Quality Measures Across Physician Group-Years (N = 2,349) eFigure 1. Consistency of Low Adjusted Performance Across Multiple Diabetes and Cardiovascular Disease Measures eFigure 2. Consistency of Low Adjusted Performance Across Multiple Disease Control and Statin Use Measures for Diabetes and Cardiovascular Disease eFigure 3. Consistency of Low Unadjusted Performance Across Multiple Measures or Multiple Years for Diabetes and Cardiovascular Disease eFigure 4. Consistency of High Adjusted Performance Across Multiple Measures or Multiple Years for Diabetes and Cardiovascular Disease eReferences. [file jamanetwopen-e2117954-s001.pdf]

Supplemental Online Content

Nguyen CA, Gilstrap LG, Chernew ME, et al. Using consistently low performance to identify low-quality physician groups. *JAMA Netw Open.* 2021;4(7):e2117954. doi:10.1001/jamanetworkopen.2021.17954

eAppendix 1. Enrollee Attribution to Tax Identification Number (TIN)

eAppendix 2. Quality Metric Coding

eAppendix 3. Two-Step Social Risk Adjustment Methodology

eTable 1. Characteristics of Enrollee-Years with Diabetes or Cardiovascular Disease by Availability of Laboratory and Pharmacy Data (%)

eTable 2. Correlations Between Adjusted Performance on Quality Measures Across Physician Group-Years (N = 2,349)

eFigure 1. Consistency of Low Adjusted Performance Across Multiple Diabetes and Cardiovascular Disease Measures

eFigure 2. Consistency of Low Adjusted Performance Across Multiple Disease Control and Statin Use Measures for Diabetes and Cardiovascular Disease

eFigure 3. Consistency of Low Unadjusted Performance Across Multiple Measures or Multiple Years for Diabetes and Cardiovascular Disease

eFigure 4. Consistency of High Adjusted Performance Across Multiple Measures or Multiple Years for Diabetes and Cardiovascular Disease

eReferences

This supplemental material has been provided by the authors to give readers additional information about their work.

© 2021 Nguyen CA et al. *JAMA Network Open.*

eAppendix 1. Enrollee Attribution to Tax Identification Number (TIN)

In each year, we attributed each enrollee to the physician group (defined by Tax Identification Number [TIN]) accounting for the plurality of the enrollee's office visits during the year: current procedural terminology [CPT] codes 99201-99215, 99241-99245, G0402, G0438, G0438, with specialty codes for family medicine [08], internal medicine [11], geriatric medicine [38], general provider organization [01] or endocrinology [46]. Enrollees with the same number of visits to more than one TIN were assigned to the TIN with the greater sum of allowed costs. TINs represent physician groups ranging from small practices to health systems.

© 2021 Nguyen CA et al. *JAMA Network Open.*

eAppendix 2. Quality Metric Coding

Testing Measures

Metric	Numerator	Denominator
**% with HbA1c Test**	Eligible enrollees with ≥1 test for HbAlc in claims during the measurement year	All eligible enrollees[a]
**% with LDL Test**	Eligible enrollees with ≥1 test for LDL in claims during the measurement year	All eligible enrollees[a]

[a] Eligible Enrollees: enrollees with a diagnosis of diabetes or CVD (≥1 inpatient or ≥2 outpatient claims for diabetes or CVD during the measurement year) and attributed to a TIN with ≥40 attributed enrollees with diabetes and ≥40 with CVD.

Disease Control Measures

Metric	Numerator	Denominator
**% with HbA1c <8%**	Eligible enrollees* whose first HbA1c value in the measurement year was <8%	Eligible enrollees[b] with ≥1 laboratory test for HbAlc (from the laboratory file) during the measurement year
**% with LDL <100mg/dl**	Eligible enrollees* whose first LDL value in the measurement year was <100mg/dl	Eligible enrollees[b] with ≥1 laboratory test for LDL (from the laboratory file) during the measurement year

[b] Eligible Enrollees: enrollees with a diagnosis of diabetes or CVD (≥1 inpatient or ≥2 outpatient claims for diabetes or CVD during the measurement year), with laboratory data, and attributed to a TIN with ≥40 attributed enrollees with diabetes and ≥40 with CVD.

Drug-Use Measures

Metric	Numerator	Denominator
**% with use of any statin**	Eligible enrollees[c] with ≥1 fill of any statin. Statin use identified in the pharmacy file using National Drug Codes (NDC)[d] during the measurement year	All eligible enrollees[c]

[c] Eligible Enrollees: enrollees with a diagnosis of diabetes or CVD (≥1 inpatient or ≥2 outpatient claims for diabetes or CVD during the measurement year), with pharmacy data, and attributed to a TIN with ≥40 attributed enrollees with diabetes and ≥40 with CVD.

[d] Statin NDC codes obtained from HEDIS 2016 and are available at: http://www.ncqa.org/hedis-quality-measurement/hedis-measures/hedis-2016/hedis-2016-ndc-license/hedis-2016-final-ndc-lists (Access Date May 21, 2018).

© 2021 Nguyen CA et al. *JAMA Network Open.*

Hospital-Based Utilization Measures

1. Any hospitalization for diabetes, including observational stays
2. Any hospitalization for major adverse cardiovascular events, including observational stays
3. Emergency department visit for diabetes (patient was not admitted)
4. Emergency department visit for major adverse cardiovascular events (patient was not admitted)

Major Adverse Cardiovascular Events (MACE) Hospital-Based Utilization Measures

Acute Coronary Syndrome: 410.x

Angina: 411.1, 411.8x, 413.x

Cerebrovascular accident/stroke: 430-432, 433-436

Malignant dysrhythmia: 427.1, 427.4, 427.41-427.42, 427.5

Sudden Cardiac Death: 798.1, 798.2

Coronary Revascularization CPT: 33510-33519, 33520-33523, 33530-33536, 92973-92984, 92995-92998

Coronary Revascularization HCPCS: S2205-S2209, G0290, G0291

Admissions/emergency department visits for diabetes were determined using the Agency for HealthCare Research and Quality's (AHRQ) prevention quality indicators (PQI 1, 3, 14 and 16) for diabetes.[1]

© 2021 Nguyen CA et al. *JAMA Network Open.*

eAppendix 3. Two-Step Social Risk Adjustment Methodology

Because low-performing groups may be more likely to treat high-risk patients, we followed a two-step social risk adjustment methodology from earlier studies[2–4] for computing adjusted quality performance. Prior work has shown that social risk adjustment can impact the variance and rankings of physician group performance on disease control and outcome measures, but has less influence on performance on process measures.[2] For consistency, we adjusted all quality measures, including process measures. In this approach, we removed the effect of high-risk patients sorting to low-quality physician groups by basing our adjustment on within-group associations. In the first step, we fit inverse probability weighted linear probability regression models with physician group fixed effects using the following model:

(1) $$q_{p,t,g} = \alpha + \beta_1' x_p + \beta_2' c_p + \beta_3' s_z + \gamma_g + \epsilon_{p,t,g},$$

where $q_{p,t,g}$ is the adherence of patient p attributed to group g to an individual quality measure in year t; x_p is a vector containing patient-level age and gender controls; c_p is patient-level comorbidities and DxCG composite; s_z is zip code-level sociodemographic characteristics; and γ_g is physician group fixed effects. We then computed an enrollee-year-level risk score as the predicted performance for each measure estimated from only the coefficients on the enrollee characteristics (i.e., not including the coefficients on the group fixed effects in the prediction) as:

(2) $$\hat{r}_{p,t} = \hat{\alpha} + \sum \hat{\beta}_1' x_p + \sum \hat{\beta}_2' c_p + \sum \hat{\beta}_3' s_z.$$

In the second step, we computed group-level performance scores. We estimated patient-level mixed effects linear probability regression models that related the performance on a measure in a given year to the risk-score computed in step 1 and physician group random effects using the following model:

(3) $$q_{p,t} = \alpha + \beta \hat{r}_{p,t} + \delta_g,$$

© 2021 Nguyen CA et al. *JAMA Network Open.*

where δ_g is physician group random effects that we assumed followed a normal distribution with variance equal to σ_g^2. We computed group adjusted performance using:

(4) $\hat{y}_{g,t} = \hat{\alpha} + \hat{\beta} r_{p,t} \mu_r + \hat{\delta}_g$

where $\hat{\delta}_g$ is the estimated random effect and μ_r is the average risk score across all enrollees. This represents a group's estimated performance for an enrollee with average clinical and social risk.

To estimate the degree of variation in performance at the physician group level, we computed intraclass correlation coefficients (ICCs):

(5) $\rho = \frac{\sigma_g^2}{\sigma_g^2 + \sigma_r^2}$

where σ_g^2 is the variance between groups and σ_r^2 is the residual variance from the linear probability model. The ICC represents the fraction of total variation in performance that is explained by differences between physician groups. The ICC can be low if there is little variation between groups or if the within group variance is high. Measures with low ICCs generally have less ability to distinguish performance at the group level and are thus less useful for identifying low-performing providers.

We also computed reliability for each measure:

(6) $reliability = \frac{\rho \eta_c}{1 + \rho(\eta_c - 1)}$

where ρ is the ICC and η_c is the median number of enrollees in a practice in a given year in the corresponding cohort *c* (diabetes or CVD). Reliability represents a measure's reproducibility and is a function of the measure's *within* and *between* practice variation and the sample size.

© 2021 Nguyen CA et al. *JAMA Network Open.*

eTable 1. Characteristics of Enrollee-Years with Diabetes or Cardiovascular Disease by Availability of Laboratory and Pharmacy Data (%)

	Diabetes				Cardiovascular Disease			
	Laboratory Data		Pharmacy Data		Laboratory Data		Pharmacy Data	
	Missing	Non-missing	Missing	Non-missing	Missing	Non-missing	Missing	Non-missing
N	252,200	299,215	315,869	235,546	406,500	301,671	416,574	291,597
Male	51.7	52.2	51.3	52.9	53.9	53.6	52.8	55.2
Age								
18-35	6.6	5.2	5.6	6.1	4.3	4.1	4.1	4.4
36-45	13.1	12.6	12.3	13.5	12.2	12.3	12.0	12.7
46-55	31.1	31.5	31.2	31.5	30.8	31.3	30.6	31.6
56-65	49.2	50.7	50.9	48.9	52.6	52.4	53.3	51.4
Atrial fibrillation	1.7	1.6	1.6	1.7	2.1	1.7	1.9	2.0
Hypertension	57.0	60.9	60.9	56.7	53.9	53.6	54.1	53.3
Chronic obstructive pulmonary disease	2.7	2.4	2.3	2.9	2.8	2.2	2.3	2.8
Heart failure	2.8	2.1	2.3	2.5	1.7	1.2	1.4	1.6
Chronic kidney disease	34.2	36.1	36.5	33.5	6.5	7.6	6.7	7.2
Zip code-level								
White	70.2	70.5	70.1	70.6	74.1	74.4	74.3	74.2
Black	15.2	14.5	15.2	14.3	12.0	11.9	12.2	11.7
Hispanic/Latino	16.6	19.1	18.0	18.0	15.3	16.6	15.7	16.1
College-educated	29.4	29.2	29.0	29.6	32.3	32.0	31.9	32.6
Below poverty	13.3	12.9	13.1	13.2	11.6	11.4	11.5	11.6
Urban	52.3	50.4	52.0	50.4	47.5	46.7	47.4	46.8
Suburban	44.5	47.5	45.3	47.2	49.7	51.0	49.8	50.9
Rural	3.2	2.1	2.8	2.4	2.9	2.2	2.8	2.3

© 2021 Nguyen CA et al. *JAMA Network Open.*

eTable 2. Correlations Between Adjusted Performance on Quality Measures Across Physician Group-Years (N = 2,349)

		Diabetes						Cardiovascular Disease		
		**HbA1c testing**	**LDL testing**	**HbA1c control**	**LDL control**	**Any statin use**	**Hospital-based utilization**	**LDL testing**	**LDL control**	**Any statin use**
	**Low-density lipoprotein testing**	-0.001								
	**Hemoglobin A1c control (<8%)**	-0.01	0.21							
	**Low-density lipoprotein control (<100 mg/dl)**	0.08	0.09	0.07						
	**Statin use**	0.02	0.01	0.03	-0.10					
	**Hospital-based utilization**	0.05	0.14	0.17	0.17	-0.11				
**Cardiovascular Disease**	**Low-density lipoprotein testing**	-0.07	0.43	0.04	-0.01	0.08	0.10			
	**Low-density lipoprotein control (<100 mg/dl)**	-0.02	0.02	-0.26	0.18	0.16	0.11	0.33		
	**Statin use**	-0.02	-0.01	-0.26	-0.08	0.55	-0.01	0.30	0.78	
	**Hospital-based utilization**	0.02	0.03	0.34	0.01	-0.22	0.07	-0.31	-0.85	-0.82

Notes: This table presents correlations for adjusted performance at the physician group-year level (pooled across 2016-2019).
Abbreviations: HbA1c = Hemoglobin A1c, LDL = Low-density lipoprotein.

© 2021 Nguyen CA et al. *JAMA Network Open.*

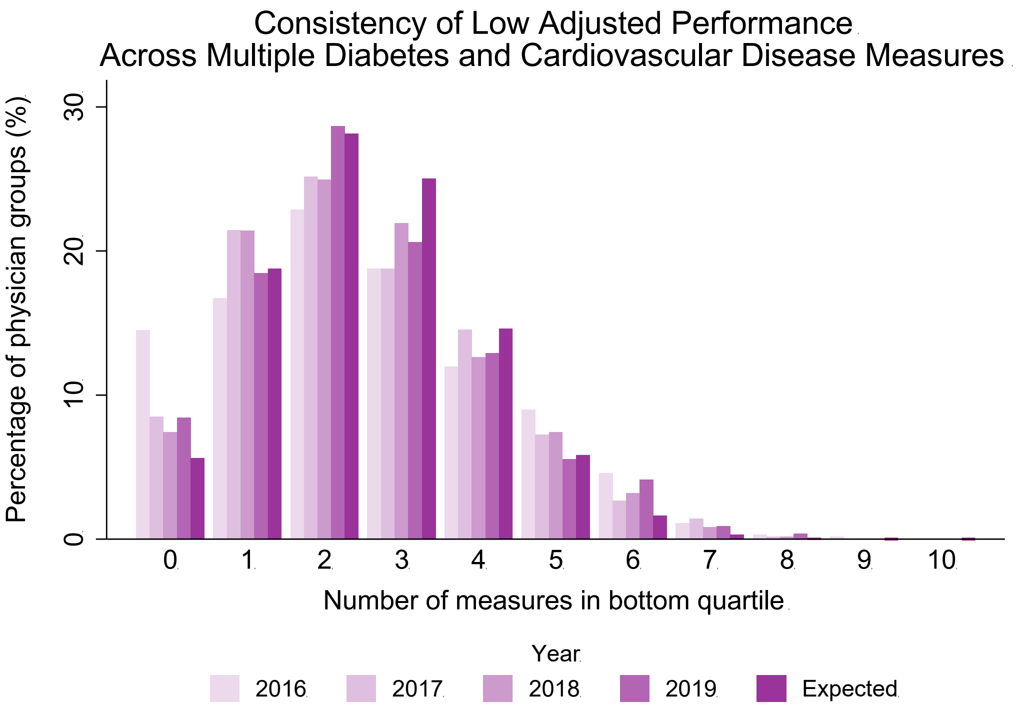

eFigure 1. Consistency of Low Adjusted Performance
Across Multiple Diabetes and Cardiovascular Disease Measures

Notes: This figure includes all ten measures for both diabetes and cardiovascular disease. The expected bar is the proportion of physician groups expected to fall into the bottom quartile if performance on each measure in a given year was independent. For example, falling into the bottom quartile for three measures was computed as the probability of three success outcomes in ten Bernoulli trials with a success probability of 0.25.

© 2021 Nguyen CA et al. *JAMA Network Open.*

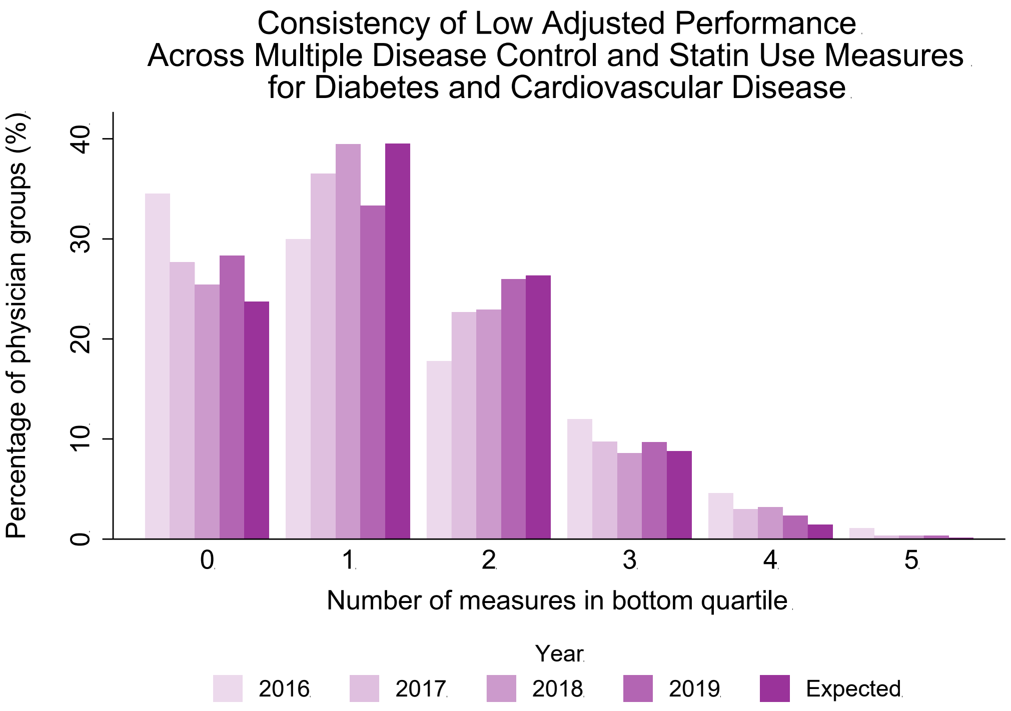

eFigure 2. Consistency of Low Adjusted Performance Across Multiple Disease Control and Statin Use Measures for Diabetes and Cardiovascular Disease

Notes: This figure includes only HbA1c control for diabetes and LDL control and statin use for both diabetes and cardiovascular disease. We excluded HbA1c testing for diabetes and LDL testing and hospital-based utilization for both diabetes and cardiovascular disease because they had overall high performance and low variation across groups. The distinction of being in the bottom quartile for those measures was less meaningful. The expected bar is the proportion of physician groups expected to fall into the bottom quartile if performance on each measure in a given year was independent. For example, falling into the bottom quartile for three measures was computed as the probability of three success outcomes in five Bernoulli trials with a success probability of 0.25.

© 2021 Nguyen CA et al. *JAMA Network Open.*

eFigure 3. Consistency of Low Unadjusted Performance Across Multiple Measures of Diabetes and Cardiovascular Disease

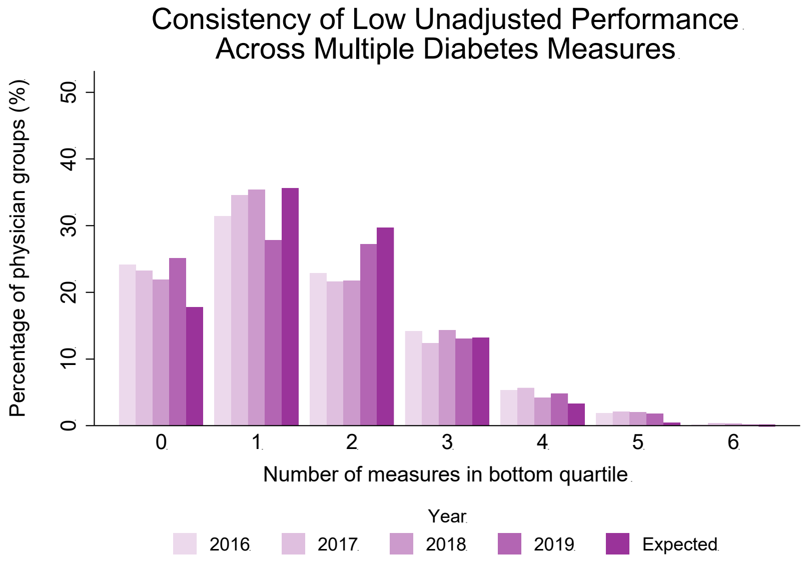

A. Consistency of Low Unadjusted Performance Across Multiple Diabetes Measures

Notes: This figure uses unadjusted quality performance (not adjusted for age, sex, or clinical or social risk factors). The expected bar is the proportion of physician groups expected to fall into the bottom quartile if performance on each measure in a given year was independent. For example, falling into the bottom quartile for three measures was computed as the probability of three success outcomes in six Bernoulli trials with a success probability of 0.25.

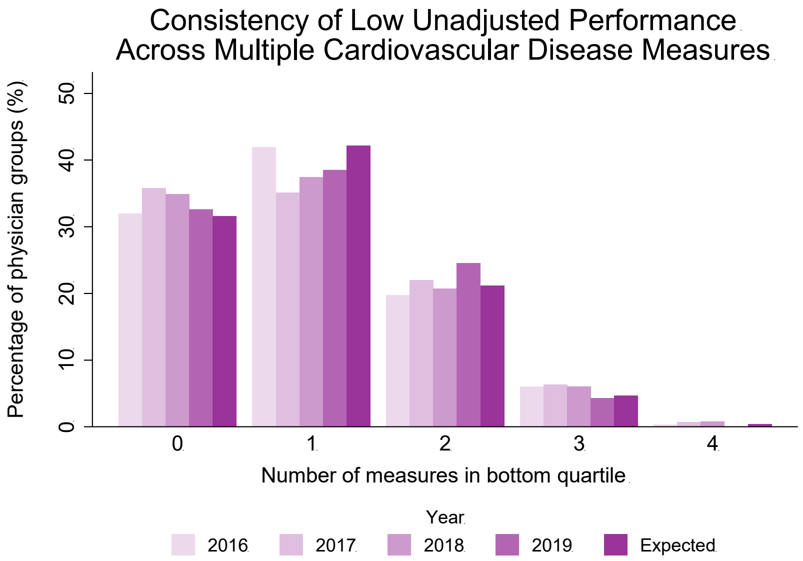

B. Consistency of Low Unadjusted Performance Across Multiple Cardiovascular Disease Measures

Notes: This figure uses unadjusted quality performance (not adjusted for age, sex, or clinical or social risk factors). The expected bar is the proportion of physician groups expected to fall into the bottom quartile if performance on each measure in a given year was independent. For example, falling into the bottom quartile for three measures was computed as the probability of three success outcomes in four Bernoulli trials with a success probability of 0.25.

© 2021 Nguyen CA et al. *JAMA Network Open.*

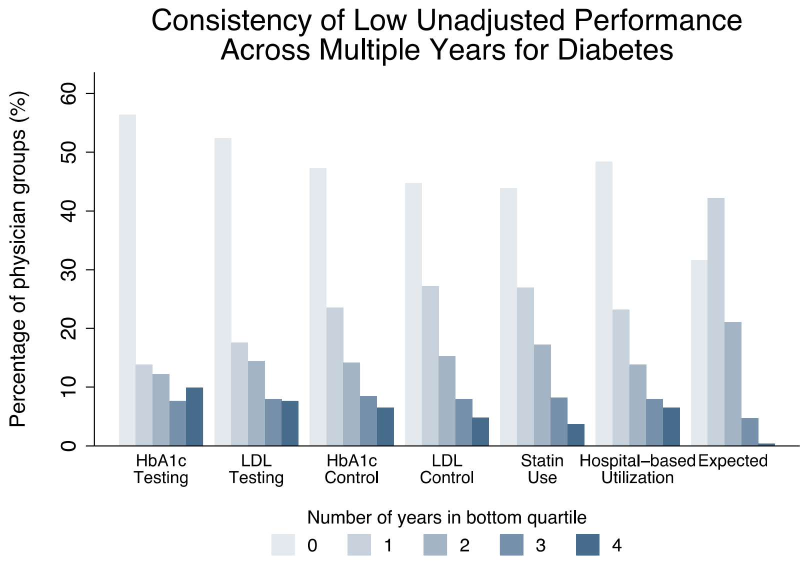

C. Consistency of Low Unadjusted Performance Across Multiple Years for Diabetes

Notes: This figure uses unadjusted quality performance (not adjusted for age, sex, or clinical or social risk factors). The expected bar is the proportion of physician groups expected to fall into the bottom quartile if performance in each year for a given measure was independent. For example, falling into the bottom quartile for three years was computed as the probability of three success outcomes in four Bernoulli trials with a success probability of 0.25. Abbreviations: HbA1c = Hemoglobin A1c, LDL = Low-density lipoprotein.

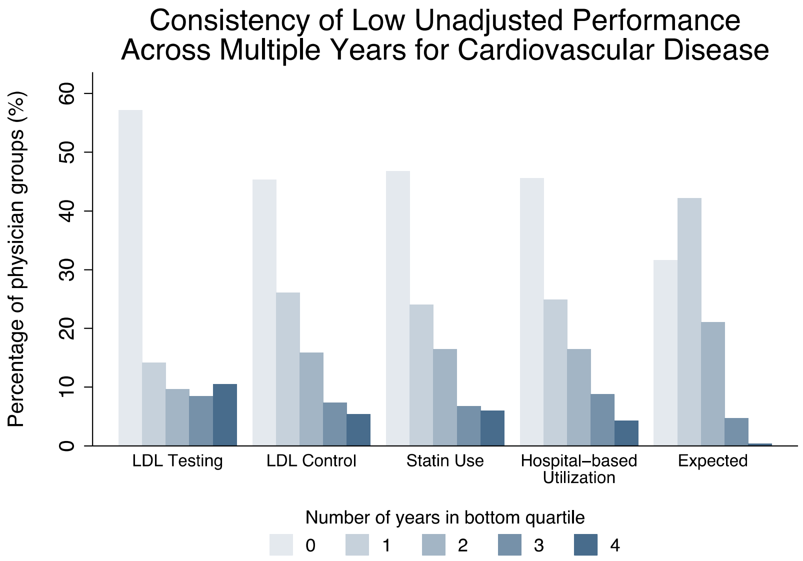

D. Consistency of Low Unadjusted Performance Across Multiple Years for Cardiovascular Disease

Notes: This figure uses unadjusted quality performance (not adjusted for age, sex, or clinical or social risk factors). The expected bar is the proportion of physician groups expected to fall into the bottom quartile if performance in each year for a given measure was independent. For example, falling into the bottom quartile for three years was computed as the probability of three success outcomes in four Bernoulli trials with a success probability of 0.25. Abbreviations: HbA1c = Hemoglobin A1c, LDL = Low-density lipoprotein.

© 2021 Nguyen CA et al. *JAMA Network Open.*

eFigure 4. Consistency of High Adjusted Performance Across Multiple Measures of Diabetes and Cardiovascular Disease

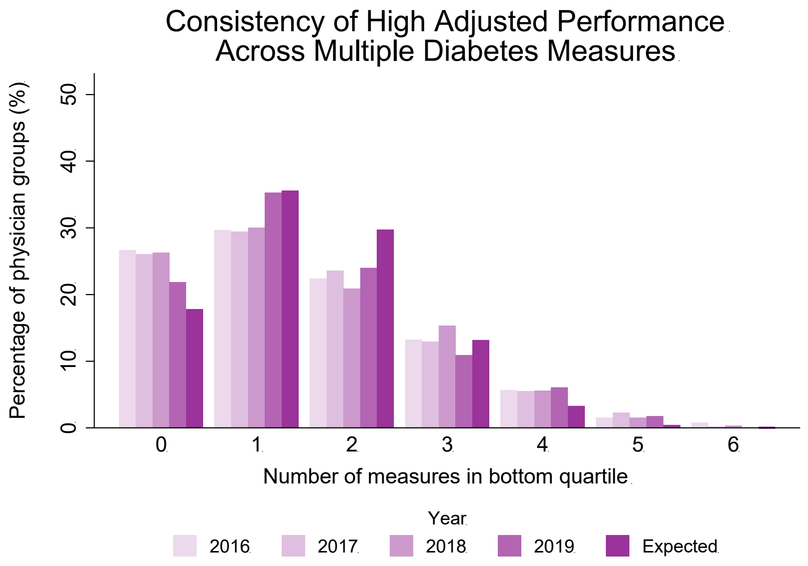

A. Consistency of High Adjusted Performance Across Multiple Diabetes Measures

Notes: High performance is defined as being in the top quartile of performance for a measure in a given year. The expected bar is the proportion of physician groups expected to fall into the top quartile if performance on each measure in a given year was independent. For example, falling into the top quartile for three measures was computed as the probability of three success outcomes in six Bernoulli trials with a success probability of 0.25.

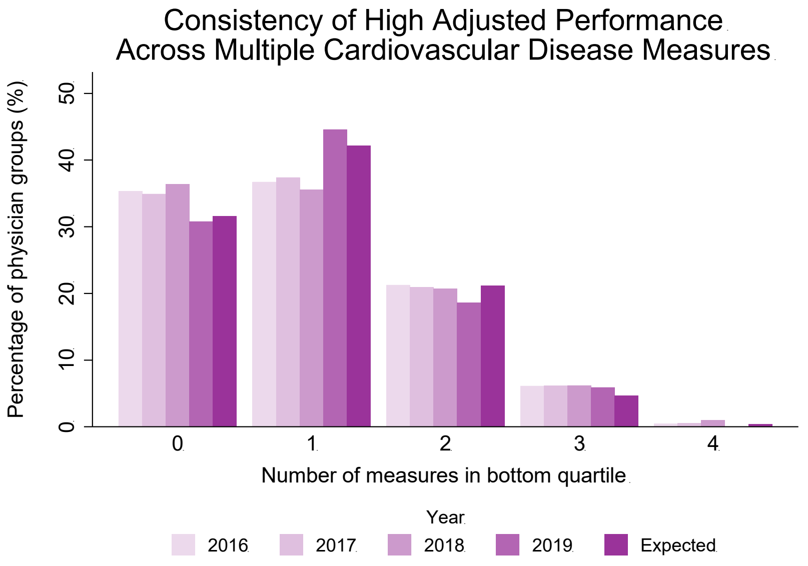

B. Consistency of High Adjusted Performance Across Multiple Cardiovascular Disease Measures

Notes: High performance is defined as being in the top quartile of performance for a measure in a given year. The expected bar is the proportion of physician groups expected to fall into the top quartile if performance on each measure in a given year was independent. For example, falling into the top quartile for three measures was computed as the probability of three success outcomes in four Bernoulli trials with a success probability of 0.25.

© 2021 Nguyen CA et al. *JAMA Network Open.*

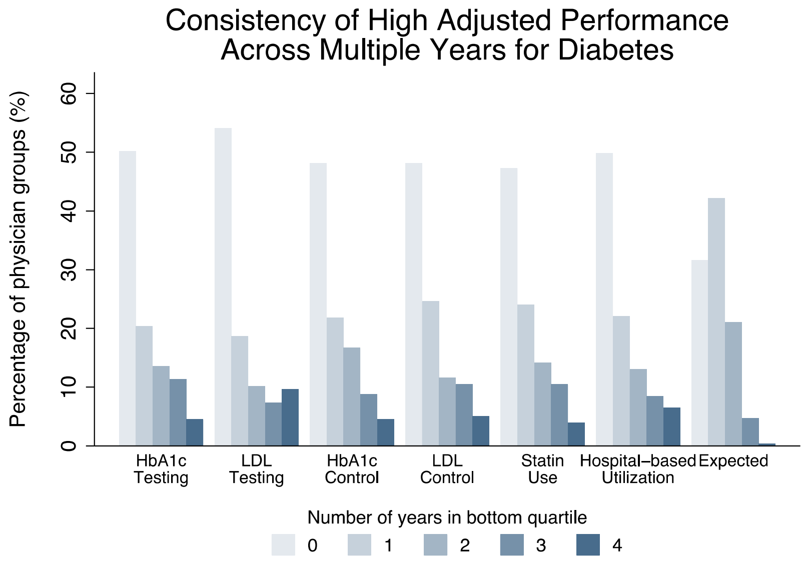

C. Consistency of High Adjusted Performance Across Multiple Years for Diabetes

Notes: High performance is defined as being in the top quartile of performance for a measure in a given year. The expected bar is the proportion of physician groups expected to fall into the top quartile if performance in each year for a given measure was independent. For example, falling into the top quartile for three years was computed as the probability of three success outcomes in four Bernoulli trials with a success probability of 0.25. Abbreviations: HbA1c = Hemoglobin A1c, LDL = Low-density lipoprotein.

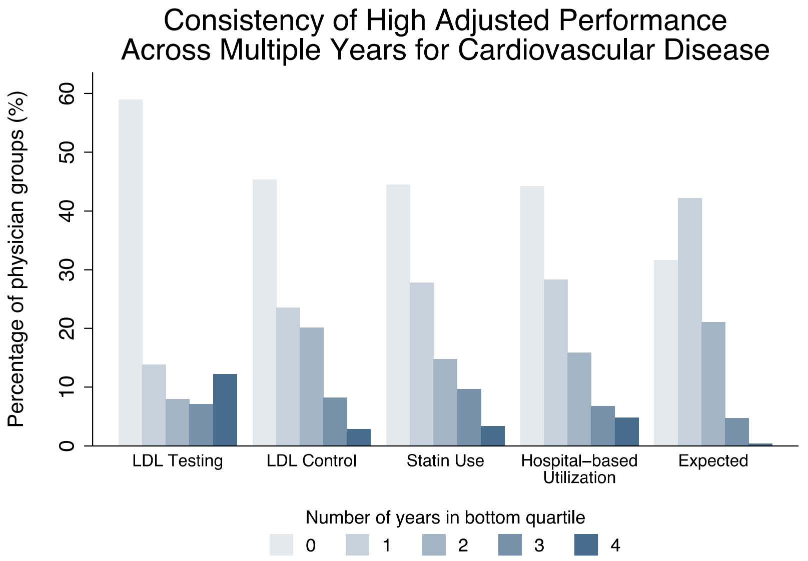

D. Consistency of High Adjusted Performance Across Multiple Years for Cardiovascular Disease

Notes: High performance is defined as being in the top quartile of performance for a measure in a given year. The expected bar is the proportion of physician groups expected to fall into the top quartile if performance in each year for a given measure was independent. For example, falling into the top quartile for three years was computed as the probability of three success outcomes in four Bernoulli trials with a success probability of 0.25. Abbreviations: LDL = Low-density lipoprotein.

© 2021 Nguyen CA et al. *JAMA Network Open.*

eReferences

1. Agency for Healthcare Research and Quality. *Prevention Quality Indicators Technical Specifications Updates - Version 6.0 (ICD-9)*.; 2016.
2. Nguyen CA, Gilstrap LG, Chernew ME, McWilliams JM, Landon BE, Landrum MB. Social Risk Adjustment of Quality Measures for Diabetes and Cardiovascular Disease in a Commercially Insured US Population. *JAMA Netw Open*. 2019;2(3):1-12. doi:10.1001/jamanetworkopen.2019.0838
3. Roberts ET, Zaslavsky AM, Mcwilliams JM. The value-based payment modifier: Program outcomes and implications for disparities. *Ann Intern Med*. 2018;168(4):255-265. doi:10.7326/M17-1740
4. Roberts ET, Zaslavsky AM, Barnett ML, Landon BE, Ding L, McWilliams JM. Assessment of the Effect of Adjustment for Patient Characteristics on Hospital Readmission Rates. *JAMA Intern Med*. 2018;02115. doi:10.1001/jamainternmed.2018.4481

© 2021 Nguyen CA et al. *JAMA Network Open.*
